# Spectroscopic characterization and crystal structures of two cathinone derivatives: 1-(4-chlorophenyl)-2-(1-pyrrolidinyl)-pentan-1-one (4-chloro-α-PVP) sulfate and 1-(4-methylphenyl)-2-(dimethylamino)-propan-1-one (4-MDMC) hydrochloride salts, seized on illicit drug market

**DOI:** 10.1007/s11419-017-0381-x

**Published:** 2017-08-29

**Authors:** Piotr Kuś, Joachim Kusz, Maria Książek, Ewelina Pieprzyca, Marcin Rojkiewicz

**Affiliations:** 1 Department of Chemistry, University of Silesia, 9 Szkolna Street, 40-006 Katowice, Poland; 2 Institute of Physics, University of Silesia, 4 Uniwersytecka Street, 40-007 Katowice, Poland; 3 Department of Forensic Medicine, Medical University of Silesia, 15 Poniatowskiego Street, Katowice, Poland

**Keywords:** 4-Chloro-α-PVP, 4-MDMC, X-ray crystallography, Infrared spectroscopy, Raman spectroscopy, NMR spectroscopy

## Abstract

**Purpose:**

Two compounds newly found in the seizures by drug enforcement agencies were identified and characterized by various instrumental analytical methods.

**Methods:**

The obtained powder samples were analyzed by gas chromatography–mass spectrometry (GC–MS), liquid chromatography–mass spectrometry^n^ (LC–MS^n^), nuclear magnetic resonance (NMR) spectroscopy, infrared and Raman spectroscopy and X-ray crystallography.

**Results:**

The two compounds were tentatively identified as 4-chloro-α-PVP and 4-MDMC by GC–MS, and LC–MS/MS. The confirmation of the results was made by NMR spectroscopy. The X-ray crystallography gave information that 4-chloro-α-PVP and 4-MDMC were in salted forms with sulfate and hydrochloride, respectively; in addition, both compounds existed as racemic mixtures.

**Conclusions:**

We could identify 4-chloro-α-PVP and 4-MDMC in the seizure powder samples by various analytical methods. X-ray crystallography was especially useful for identifying the salted forms and enantiomeric forms.

**Electronic supplementary material:**

The online version of this article (doi:10.1007/s11419-017-0381-x) contains supplementary material, which is available to authorized users.

## Introduction

Crystallographic analysis is not included routinely among techniques used for analyzing chemical compounds, and certainly not for those appearing on “designer drug” market or for narcotics. A few papers have reported X-ray crystallographic structures of cathinones [1–6]. Recently, α-pyrrolidinophenones have started to appear on the market of novel psychoactive substances (NPS) [7, 8]; the characteristics of these compounds have been reviewed [7]. It could be reasonably expected that various modifications of these compounds, with unknown biological action and potentially health- or life-threatening effects, will appear on the market of psychoactive substances.

Quite often, seizures by police contain crystalline substances. In such cases crystallographic analysis allows quick identification of the investigated compound. However, one condition should be met: the crystallographic database must already contain the correponding data of the given compound. Therefore, it is important to supply such a data for as many compounds as possible in order to allow researchers to identify compound(s) based on the unit cell parameters. Such parameters can be determined quickly and precisely without detailed compound analysis. Investigation of this type of material is possible even for mixtures found on the illicit drug market. Such compounds often can be easily crystallized from solutions used, for example, during nuclear magnetic resonance (NMR) analysis.

During spectroscopic and crystallographic investigation of compounds found on the illicit designer drug market, we encountered two compounds belonging to the cathinone category.

**Fig. 1:**
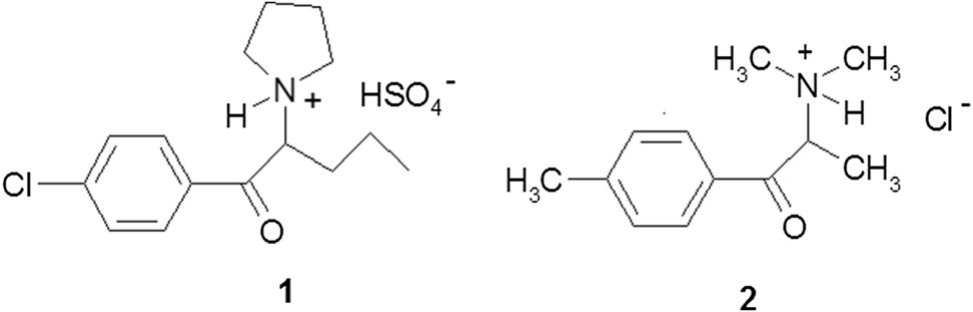
Structures of 1-(4-chlorophenyl)-2-(1-pyrrolidinyl)-pentan-1-one sulfate salt (**1)** and 1-(4-methylphenyl)-2-(dimethylamino)-propan-1-one hydrochloride (**2)**

The two compounds described herein as the materials seized by drug enforcement agencies were examples of emerging NPS appearing on the illicit drug market. Their structures are shown in Fig. 1. They are marketed as almost pure crystalline substances and also as mixtures with other substances.

In this study, we present identification of the two compounds in seized materials by gas chromatography–mass spectrometry (GC–MS) and liquid chromatography–mass spectrometry^n^ (LC–MS^n^) and various corroborative data, such as those by nuclear magnetic resonance (NMR) spectroscopy, infrared (IR) and Raman spectroscopy, and X-ray crystallography. The X-ray crystallographic analysis can give information on the salted form(s) and optical isomeric form(s) of the target compounds.

## Materials and methods

Liquid and gas chromatography reagents were of the high-performance liquid chromatography and mass spectrometry grade and were purchased from Sigma-Aldrich (Poznań, Poland). For the NMR analysis, deuterated chloroform (CDCl_3_) and dimethyl sulfoxide (DMSO-*d*_*6*_) were also purchased from Sigma-Aldrich.

1-(4-Chlorophenyl)-2-(1-pyrrolidinyl)-pentan-1-one (4-chloro-α-PVP, **1**) sulfate and 1-(4-methylphenyl)-2-(dimethylamino)-propan-1-one (4-MDMC, **2**) hydrochloride salts were provided by drug enforcement agencies as materials of seizures on the illicit drug market, either in pure form (compounds **1** and **2)** or mixtures of crystals with more than eightfold excess of taurine (according to NMR data of the second sample of compound **2**). Crystals of both compounds suitable for crystallography were obtained by very slow evaporation of DMSO-*d*_6_ or CDCl_3_ solutions used in NMR studies. ^1^H NMR spectra were recorded in CDCl_3_ or DMSO-*d*_*6*_ using a Bruker spectrometer (400 MHz) (Bruker, Bremen, Germany). The peaks were referenced to the residual CDCl_3_ (7.28 and 77.04 ppm) and DMSO-*d*_6_ (2.49 and 39.5 ppm) resonances in ^1^H and ^13^C NMR. The IR spectra were recorded on the Nicolet iS50 FT-IR Spectrometer (Thermo Scientific, Warsaw, Poland), using the attenuated total reflection technique. Raman measurements were made using a Thermo Scientific™ DXR™2xi Raman imaging microscope, and the data were collected using a 780 nm laser. LC–MS analysis of samples was performed on a Thermo LCQ DecaXP-Plus mass spectrometer, equipped with an electrospray ionization (ESI) interface (Thermo Scientific) operating in the positive mode. Separation was achieved using a Hypersil RP C18 (150 × 4.6 mm, i.d.) column (Thermo Scientific) maintained at 25 °C. Mobile phase A was 0.02 M formic acid and 0.05 M ammonium formate in water and phase B was 10% of solvent A and 90% of acetonitrile. A gradient program was applied. Product ion formation in ESI-MS^2^ and ESI-MS^3^ mode was recorded at collision energies of 30 and 35%, respectively, in the range between *m*/*z* 50 and 500. The source temperature was 250 °C, and the carrier and ionizing gases were nitrogen and helium, respectively. The obtained data were processed with Xcalibur and LCQTune programs (Thermo Scientific).

Gas chromatography–mass spectrometry analyses were performed using a Thermo Trace Ultra chromatograph coupled to a mass spectrometer (Thermo DSQ; Thermo Scientific) using an Rxi^®^-5SiL MS column (30 m length, 0.25 mm inner diameter, 0.25 µm film thickness; Restek, Bellefonte, PA, USA). Helium was used as a carrier gas at the flow rate of 1.2 mL min^−1^. The temperature program was: the initial column temperature (100 °C) was maintained for 1 min, and then increased linearly at 20 °C min^−1^ up to 260 °C. The mass detector was set to positive electron ionization (EI) mode, and the electron beam energy was 70 eV. The mass detector was operating in a full scan mode in the range of *m*/*z* 40–450. Injection volume was 1 μL on splitless mode.

Differential scanning calorimetry (DSC) was performed with a TA-DSC 2010 (TA Instruments, New Castle, DE, USA) using aluminum sample pans. The DSC experiments were carried out in a nitrogen atmosphere with a temperature range from 25 °C to over the melting point.

Single crystal X-ray experiments were performed at 100 K (**1**) or 293 K (**2**). The data were collected using a SuperNova kappa diffractometer with Atlas CCD detector (Agilent Technologies, Santa Clara, CA, USA) and an Xcalibur diffractometer with a Sapphire3 CCD detector (Oxford Diffraction, Sevenoaks, UK), respectively. In both cases MoKα radiation was used (*λ* 0.71073 Å). Collected data were integrated with CrysAlis Pro software (version 1.171.38.41q for **1**, 1.171.38.43f for **2**; Rigaku Oxford Diffraction 2015). The solving and refining procedures were similar for both compounds. The structures were solved using direct methods with the SHELXS-2013 software and the solutions were refined using SHELXL-2014/7 program [9]. CCDC 1548699 and CCDC 1548700 contain supplementary crystallographic data for this paper. These data can be obtained free of charge from The Cambridge Crystallographic Data Centre via http://www.ccdc.cam.ac.uk/data_request/cif.

## Results and discussion

### GC–MS and LC–MS^n^

Figure S1 shows GC–EI-MS mass spectra of compounds **1** and **2**. They agreed with those of 4-chloro-α-PVP and 4-MDMC of the described data [10–13].

Figures S2–S4 show LC–ESI-MS^n^ spectra of both compounds. The ESI-MS^2^ spectra (Figs. S3, S4) supported the identities as 4-chloro-α-PVP and 4-MDMC [12, 13].

### NMR spectra

Figures S5 and S6 show NMR spectra of compounds **1** and **2**, respectively. Both spectra demonstrated that the examined substances were chemically pure. The N–H proton of compound **1** appeared at *δ* = 10 ppm, and for the N–H proton of compound **2** at *δ* = 12.5 ppm (Table S1). The NMR spectra of both compounds confirmed the presence of di-substituted (*para*) benzene rings. The methinic protons appeared as a broad singlet (at *δ* = 5.49 ppm) for compound **2**, and as a quartet (at *δ* = 5.30 ppm) for compound **1**. The pyrrolidine unit protons of compound **1** appeared at 3.61, 3.49, 3.28, 3.05 and 2.25–1.75 ppm as multiplets. Two *N*-methyl groups of compound **2** yielded two chemically inequivalent broad singlets at 3.08 and 2.98 ppm; *para*-methyl from phenylene unit appeared as a singlet at *δ* = 2.42 ppm.

The ^13^C NMR spectra displayed carbonyl resonance at 196.3 and 195.2 ppm for compounds **1** and **2**, respectively (Table S1). The signals of aromatic carbons appeared in typical positions. There were small shifts between four signals of both compounds which reflect chemical and structural differences. The methinic carbons resonated at 68.2 and 62.2 ppm for compounds **1** and **2**, respectively. Both *N*-methylenes of pyrrolidine unit of compound **1** appeared as two peaks at 54.6 and 52.4 ppm, and both other methylenes appeared as singlets at 23.2 ppm. Similarly, both *N*-methyl groups of compound **2** resonated at 42.2 and 37.9 ppm. These NMR results are generally in accord with those of the previous studies with deuterated methanol as solvent [12, 13].

### IR and Raman spectra

In the case of analysis of unknown compounds—this often being the case with designer drugs—IR oscillation spectroscopy and Raman spectra are good techniques for corroborating the structure of an unknown substance. Both techniques can be useful in characterizing a compound, especially when we deal with isomeric compounds’ analysis. The Raman spectra of a few cathinones were discussed in [14]. In that paper the authors employed HgCl_2_ to identify mephedrone isomers.

Most characteristic in the IR spectra of both compounds were the C=O absorptions (Figs. S7, S8). They appear at 1688 and 1681 cm^−1^ for **1** and **2**, respectively. The other important bands are the aromatic ring vibrations. They appeared at 1588 and 1605 cm^−1^ for **1** and **2**, respectively. Similarly, very strong frequencies were observed for both compounds in the Raman spectra; the C=O absorptions appeared at 1690 and 1684 cm^−1^, and aromatic ring vibrations appeared at 1590 and 1606 cm^−1^ for **1** and **2**, respectively. Intensities of C=O absorption were higher than aromatic ring absorptions in IR spectra. In Raman spectra the opposite was true: aromatic ring absorptions were higher than C=O absorptions for both compounds. The IR frequencies at 1161 and 1155 cm^−1^ can be associated with tertiary amine molecular fragment of **1** and **2**, respectively. The IR spectra were also reported in the context of institutions [10, 11].

### Melting points

Differential scanning calorimetry and hot-plate analysis showed that the melting points of compound **1** were 213 and 208–211 °C with decomposition, respectively. The melting point of compound **2** was 204 °C with decomposition. The hot-plate analysis showed that the melting started at 186 °C and finally melted at 195 °C.

### X-ray crystallography

4-Chloro-α-PVP (**1**) formed monoclinic crystals in the P2_1_/n space group. 4-MDMC (**2**) formed triclinic crystals in the P-1 space group. Both compounds occurred in the examined crystals as paired enantiomers.

**Fig. 2:**
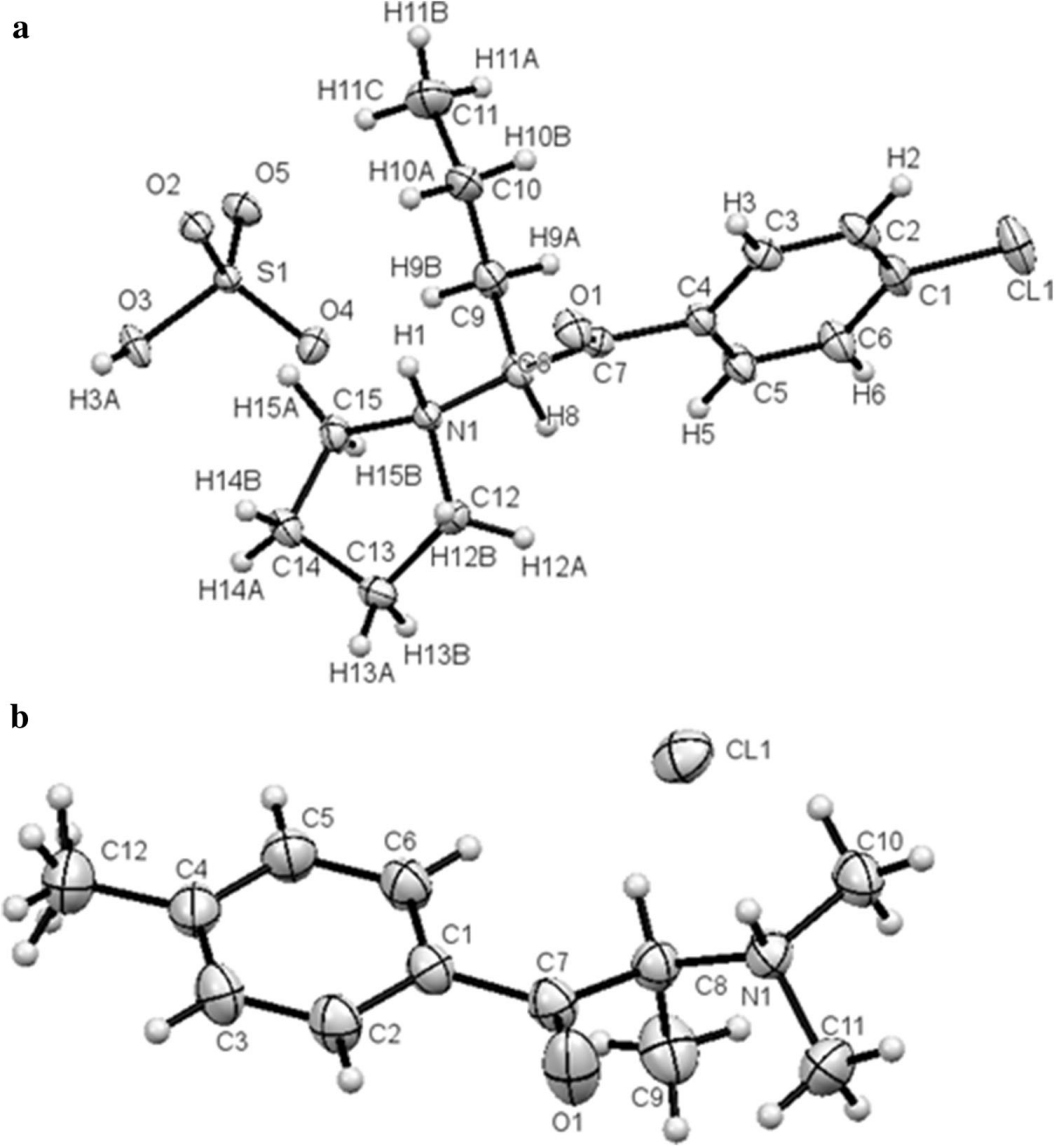
Molecules of an (S) enantiomers of **1** (**a**) and **2** (**b**) in the crystals. *Ellipsoids* correspond to 50% probability levels

**Fig. 3:**
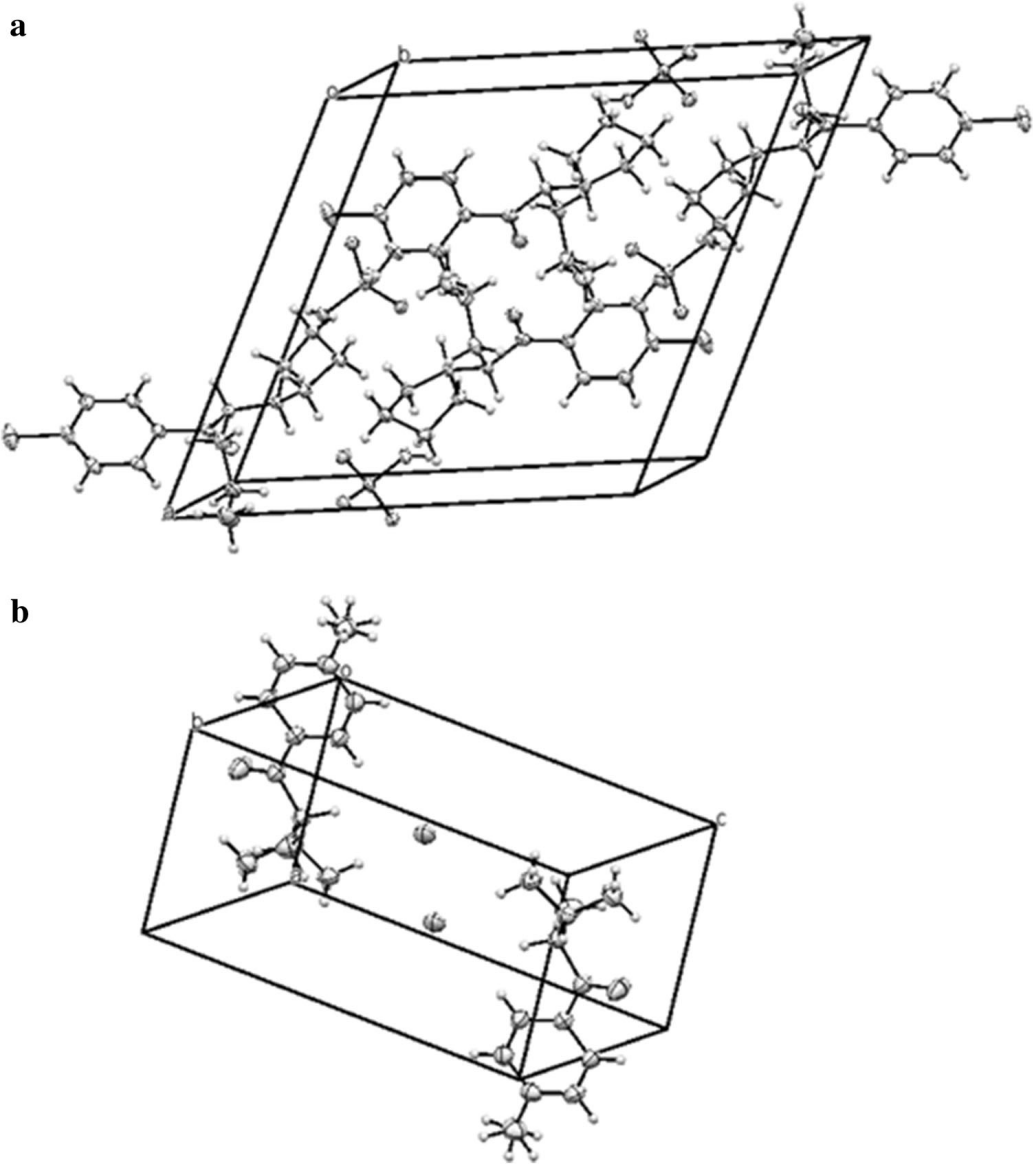
Packing diagram of **1** (**a**) and **2** (**b**)

Crystal data and structure refinement for both compounds are summarized in Table 1. The molecular structures of both compounds are shown in Fig. 2. Packing diagrams for both compounds are shown in Fig. 3. The ring systems in both compounds were planar. All distances and angles in the molecular structures were typical.

**Table 1:** Crystal data and structure refinement for compounds **1** and **2**

	Compound **1**	Compound **2**
Molecular formula	C_15_H_22_ClNO_5_S	C_12_H_18_NOCl
Molecular weight	363.84	227.72
Crystal system	Monoclinic	Triclinic
Space group	P2_1_/n	P-1
Temperature (K)	100	293
*a* (Å)	13.9389(2)	7.0754(2)
*b* (Å)	9.38130(10)	7.4206(2)
*c* (Å)	14.6723(3)	12.2153(4)
*α* (^o^)	90	105.421(3)
*β* (^o^)	115.904(2)	90.082(2)
*γ* (^o^)	90	95.107(2)
*V* (Å^3^)	1725.85(5)	615.59(3)
*Z*	4	2
Dx (g cm^−3^)	1.400	1.229
Absorption coeff. (mm^−1^)	0.37	0.286
*F* (000)	768	244
Radiation type	MoKα	MoKα
Crystal size (mm)	0.52 × 0.26 × 0.09	0.504 × 0.190 × 0.180
Data collection and structure solution		
Data collected	16,504	13,954
Independent reflections	3531	4670
Observed reflections [*I* > 2*σ*(*I*)]	3257	3627
*R* (int.)	0.030	0.022
Completeness (%)	99.8	97.9
*T*_max_/*T*_min_	1.000/0.419	1.000/0.991
No of parameters	210	141
R1 [*I* > 2*σ*(*I*)]	0.042	0.0390
wR2 (all data)	0.115	0.1080
S	1.05	1.030
Largest difference peak and hole (eÅ^−3^)	0.93, −0.63	0.27, −0.22

#### Compound **1**

In the crystals of 1-(4-chlorophenyl)-2-(1-pyrrolidinyl)-pentan-1-one (**1**) sulfate, a racemic mixture was present. The compound had two enantiomeric ion pairs in the unit of the crystal lattice (Fig. 3). The torsion angles C7C8N1C15 were identical in both enantiomers (170.75(14)°).

The cathinone molecules were mutually linked by hydrogen bonds formed with hydrogen sulfate ions occurring between these molecules.

All short contacts were linked to the oxygen atoms of hydrogen sulfate ions, the ammonium group of pyrrolidinyl units and the fourth methylene group in pentyl moiety. Distances between N–H, as well as C–H, and oxygens of hydrogen sulfate ion were 1.915 and 2.431Å, respectively. Additionally there are C–H···π interactions (3.594 Å, angle 164.8°) between the C5–H and the centroid of the phenyl ring of a neighbor molecule.

The sulfuric acid was singly deprotonated to hydrogen sulfate (HSO_4_^−^). There were four cathinone cations and four hydrogen sulfate anions in the crystal cells. Anions formed pairs of molecules with short hydrogen bonds (ca. 1.771Å). We did not observe any hydrogen bonded chains as compared to the crystal structure of mephedrone hydrogen sulfate described previously [5].

The sulfate anions adopted a tetrahedral configuration with their angles from 104.36(8)° to 112.51(8)° and S–O bond distances: from 1.442(1) to 1.466(1) Å for three bonds and 1.571(1) Å for bond in S–O–H unit.

#### Compound **2**

In the crystals of 4-MDMC (**2**) hydrochloride, a racemic mixture was present. The compound had two enantiomeric molecules in the unit of the crystal lattice (Fig. 3). Torsional angles C7C8N1C10 were identical in both enantiomers (160.90(9)°).

The chloride ions incorporated in the crystal structure of compound **2** play a major role in their stabilization. The strong chloride acceptors were bound to one strong N–H donors (N···Cl = 3.031 Å, N–H···Cl = 2.135 Å, N–H···Cl = 151.15°) and four C–H weak donors (C–H···Cl = 2.743, 2.797, 2.931 and 2.947 Å, respectively; 152° < N–H···Cl angle <173°).

## Conclusions

The testing of illicit designer drugs requires more reliable techniques for analyzing the composition of police-seized suspected substances. One such technique is crystallographic analysis, which allows unequivocal determination of the composition of single crystals present in mixtures of various compounds. This technique requires access to crystallographic databases. Our report is linked to the CCDC repository entry of the compound, where its characteristic data can be found, including elementary cell data particularly useful in quick analysis. In order to facilitate analysis of narcotics, the crystallographic databases need to be furnished with data for substances available in the drug market (not legally admitted or that will not be admitted in the future), which pose dangerous health effects for their users. In this report, we described the structures of two marketed designer drugs (4-chloro-α-PVP and 4-MDMC) and their full characteristics, using various analytical techniques. Among the techniques, X-ray crystallographic analysis was stressed, because it gives information on a compound's enantiomeric situation and the identity of a salt stabilizing the crystal. The presented results could prove helpful in analyzing unknown samples.

## Electronic supplementary material

Below is the link to the electronic supplementary material.

Supplementary material 1 (PDF 665 kb)
