# Supplementary material for: Spectroscopic characterization and crystal structures of two cathinone derivatives: 1-(4-chlorophenyl)-2-(1-pyrrolidinyl)-pentan-1-one (4-chloro-α-PVP) sulfate and 1-(4-methylphenyl)-2-(dimethylamino)-propan-1-one (4-MDMC) hydrochloride salts, seized on illicit drug market
Source: Forensic Toxicol. 2017 Aug 29;36(1):178–84. doi: 10.1007/s11419-017-0381-x (PMC5754379; doi:10.1007/s11419-017-0381-x)
Supplement: Supplementary file 1 — Supplementary material 1 (PDF 665 kb) [file 11419_2017_381_MOESM1_ESM.pdf]

**Forensic Toxicology**

# Spectroscopic characterization and crystal structure of two cathinone derivatives: 1-(4-chlorophenyl)-2-(1-pyrrolidynyl)pentan-1-one (4-chloro-α-PVP) hydrogen sulfate and 1-(4-methylphenyl)-2-(dimethylamino)propan-1-one (4-MDMC) hydrochloride, seized on illicit drug market.

Piotr Kuś[1], Joachim Kusz, Maria Książek, Ewelina Pieprzyca, Marcin Rojkiewicz

[1] Department of Chemistry, University of Silesia, 9, Szkolna Street, 40-006 Katowice, Poland

*E-mail address:* pkus@ich.us.edu.pl (P. Kuś)

**Table S1**. NMR data of compounds **1** and **2** in DMSO-$d_6$ recorded at 400 MHz ($^1$H) and 100.5 MHz ($^{13}$C), respectively

(structures of compounds **1** and **2** with the numbering of carbon atoms)

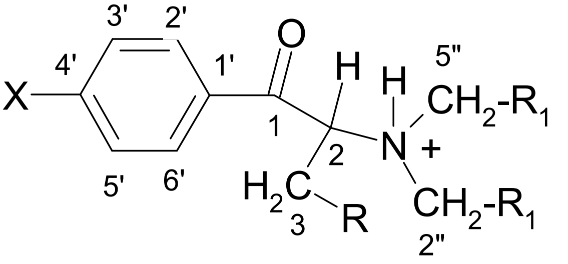


**1** R = - $CH_2$-$CH_3$ (4, 5), $R_1$ - $R_1$ = -$CH_2$-$CH_2$- (3", 4"), X = Cl

**2** R = H, $R_1$ = H, X = -$CH_3$ (7')

| Position | Compound **1** | | Compound **2** | |
|---|---|---|---|---|
| | $^1$H NMR δ(ppm), J (Hz) | $^{13}$C NMR δ(ppm) | $^1$H NMR δ(ppm), J (Hz) | $^{13}$C NMR δ(ppm) |
| 1 | | 196.3 | | 195.2 |
| 2 | 5.30 (q, *J*=7.3, 1H) | 68.2 | 5.49 (bs, 1H) | 62.2 |
| 3 | 1.96 (m, 2H) | 32.2 | 1.75 (d, *J*=1.75, 3H) | 21.8 |
| 4 | 1.96 (m, 2H) | 17.5 | | |
| 5 | 0.78 (t, 3H) | 14.1 | | |
| 1’ | | 140.6 | | 131.3 |
| 2’/6’ | 8.12 (d, *J*=8.6, 2H) | 129.9 | 7.87 (d, *J*=8.3, 2H) | 128.8 |
| 3’/5’ | 7.71 (d, *J*=8.6, 2H) | 131.3 | 7.31 (d, *J*=8.3, 2H) | 130.0 |
| 4’ | | 133.3 | | 146.4 |
| 7’ | | | 2.42 (s, 1H) | 15.8 |
| 2” | 3.61, 3.49 (2bs, 2H) | 54.6 | 3.08, 2.98 (2bs, 6H) | 37.9, 42.2 |
| 3” | 2.04 (m, 2H) | 23.2 | | |
| 4” | 1.19, 1.01 (2m, 2H) | 23.2 | | |
| 5” | 3.28, 3.05 (2bs, 2H) | 52.4 | | |
| N-H | 10.1 (bs, 1H) | | 12.5 (bs, 1H) | |

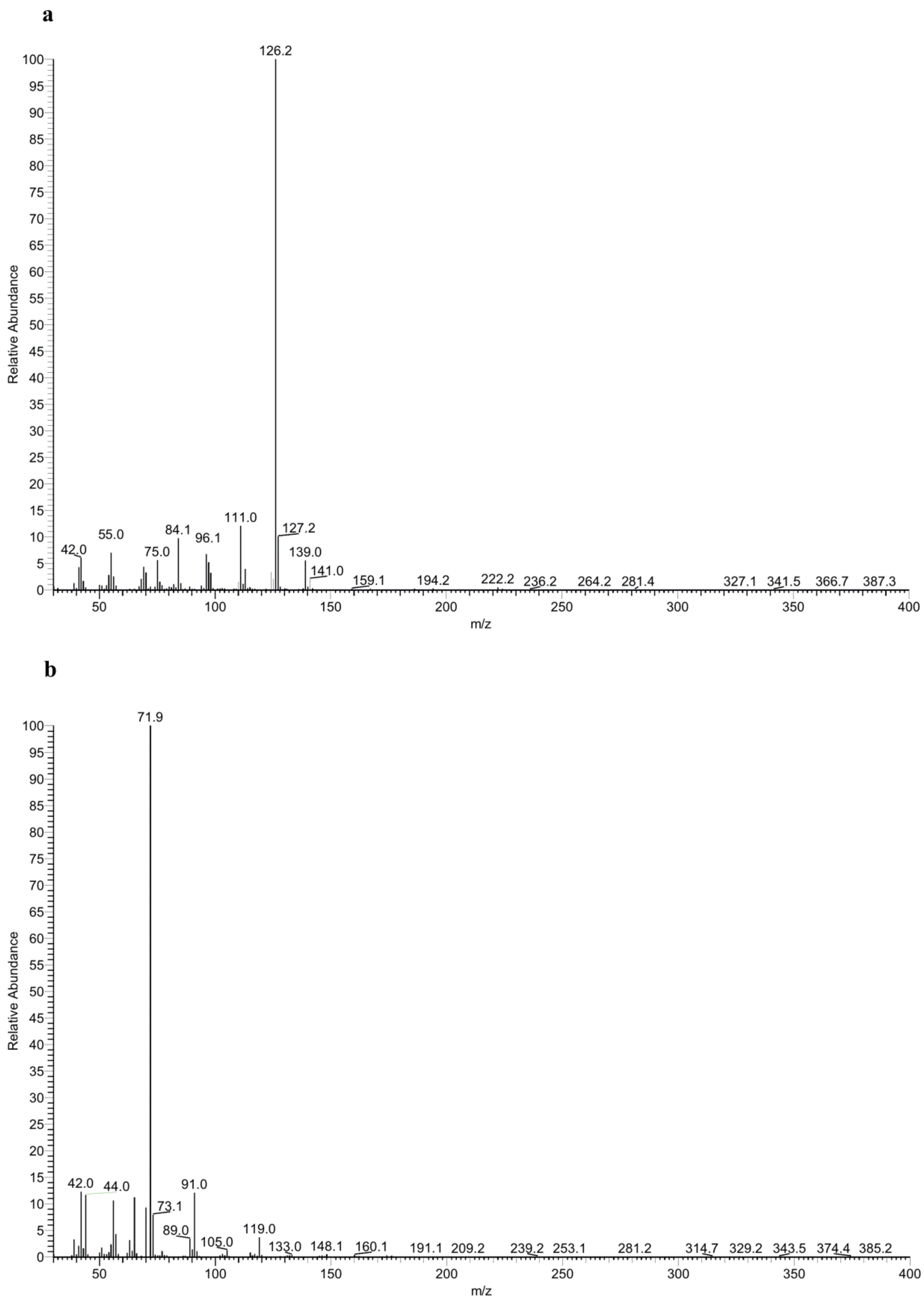


**Fig.S1** Electron ionization (EI) mass spectra of: **a** compound **1** and **b** compound **2**

**a**

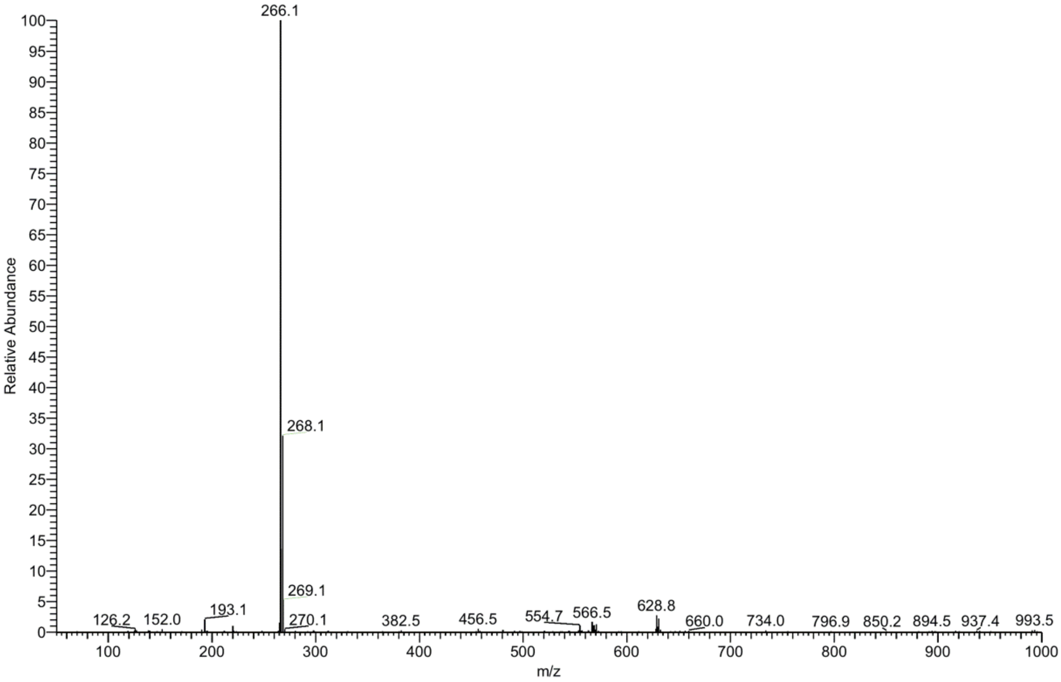


**b**

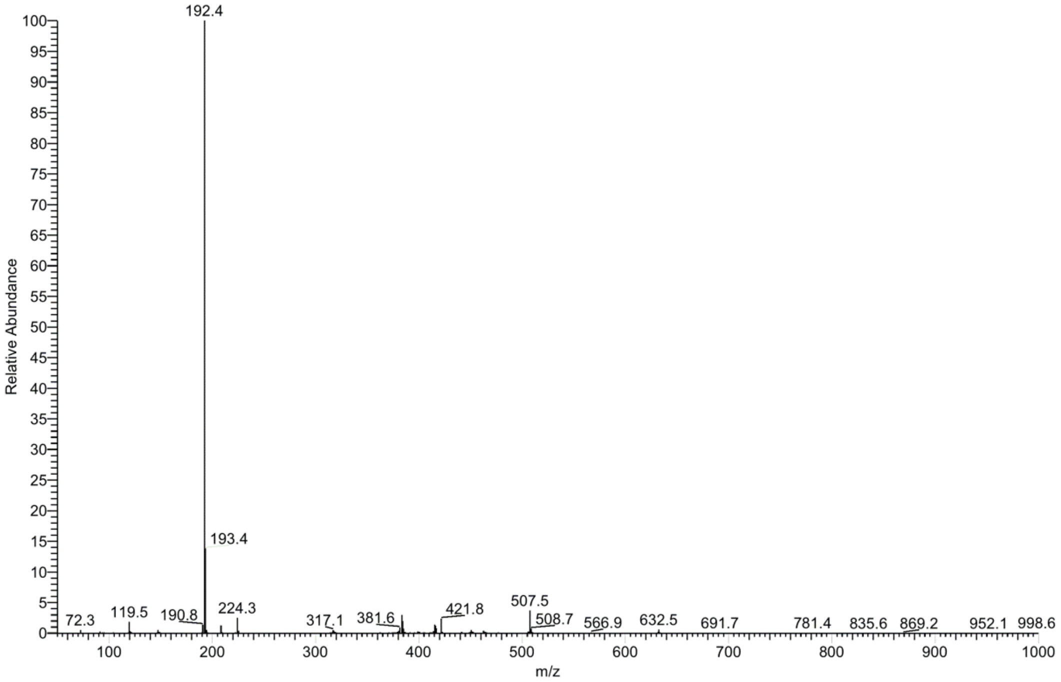


**Fig.S2** Electrosprey ionization (ESI) mass spectra of **a** compound **1** and **b** compound **2**

**a**

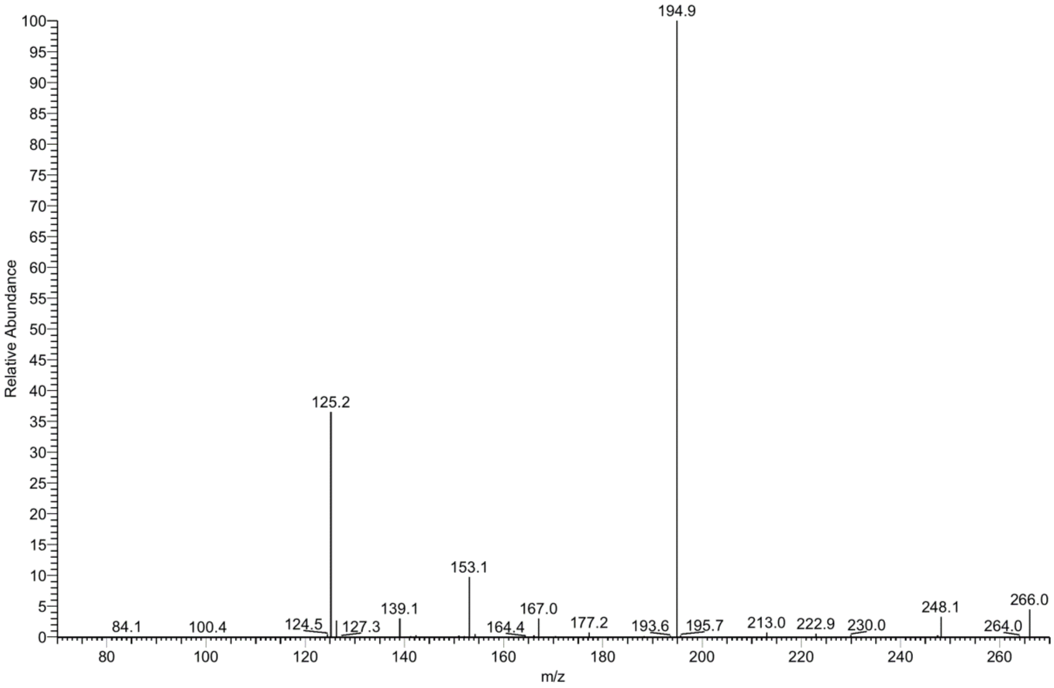


**b**

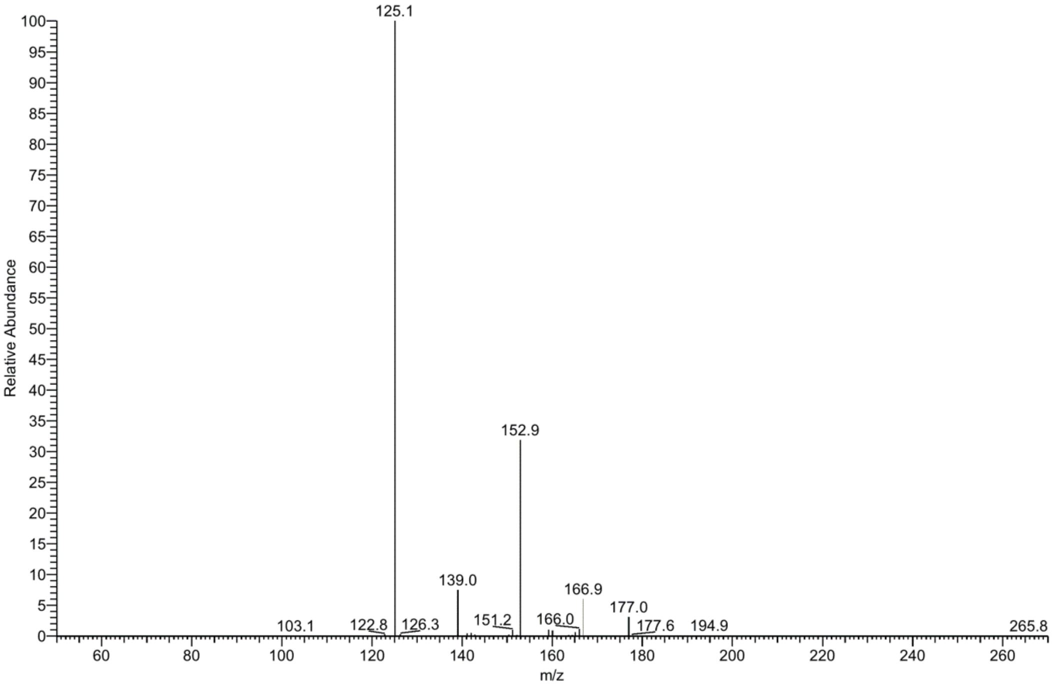


**Fig. S3** The ion mass spectra obtained by the ion trap mass spectrometry in the $MS^2$ (**a**) and $MS^3$ (**b**) mode for compound **1**. The precursor ions were used for the $MS^2$ and $MS^3$ modes: at *m/z* 266 and 195, respectively

**a**

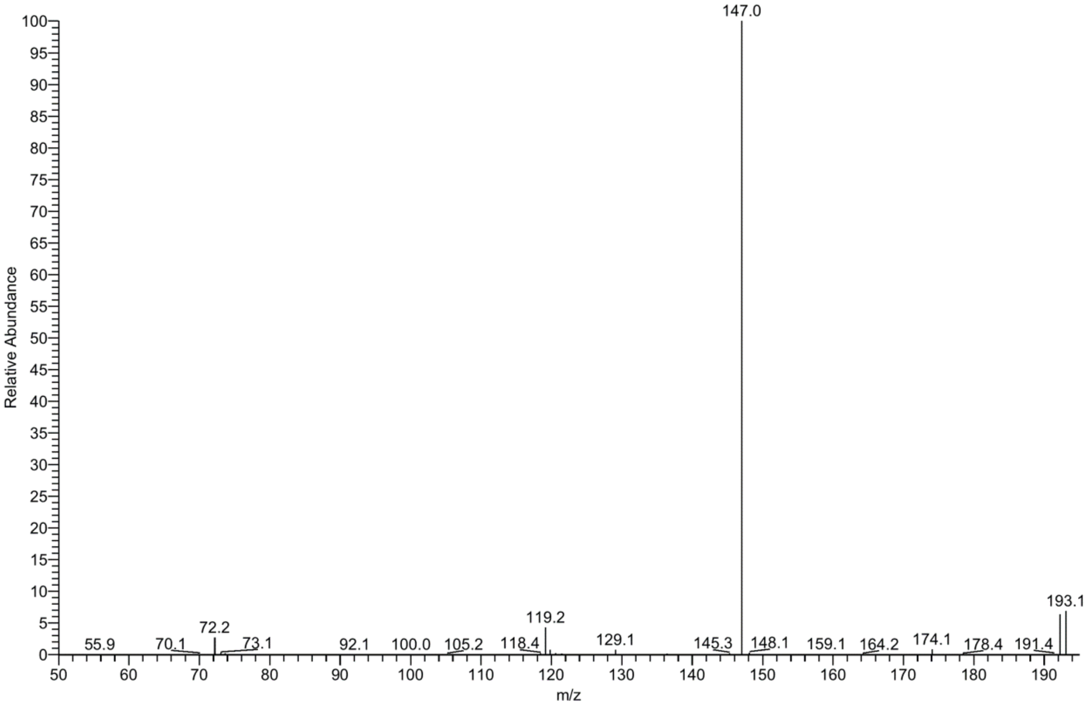


**b**

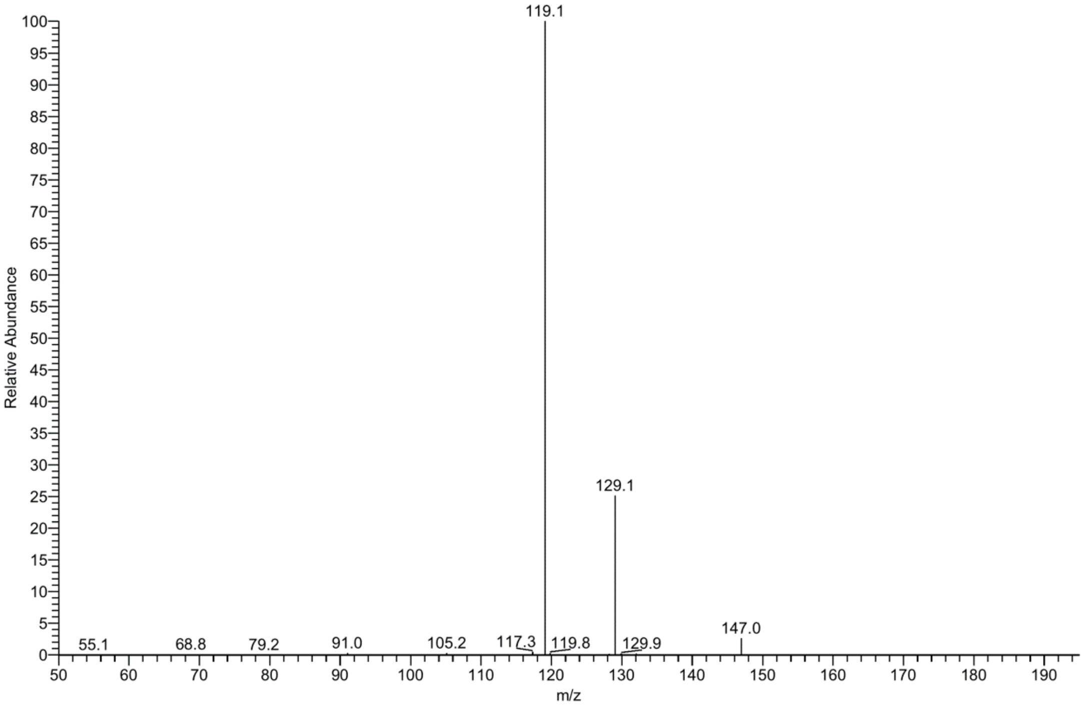


**Fig. S4** The ion mass spectra obtained by the ion trap mass spectrometry in the $MS^2$ (**a**) and $MS^3$ (**b**) mode for compound **2**. The precursor ions were used for the $MS^2$ and $MS^3$ modes: at *m/z* 192 and 147, respectively

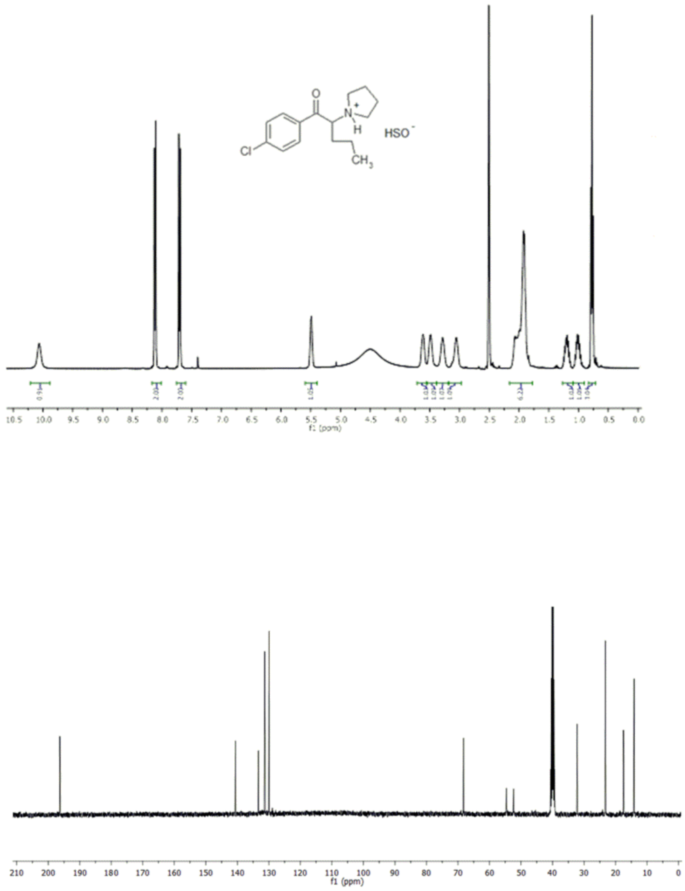


**Fig. S5** $^{1}$H NMR ( *upper*) and $^{13}$C NMR (*lower*) spectra of compound **1** in DMSO-$d_6$

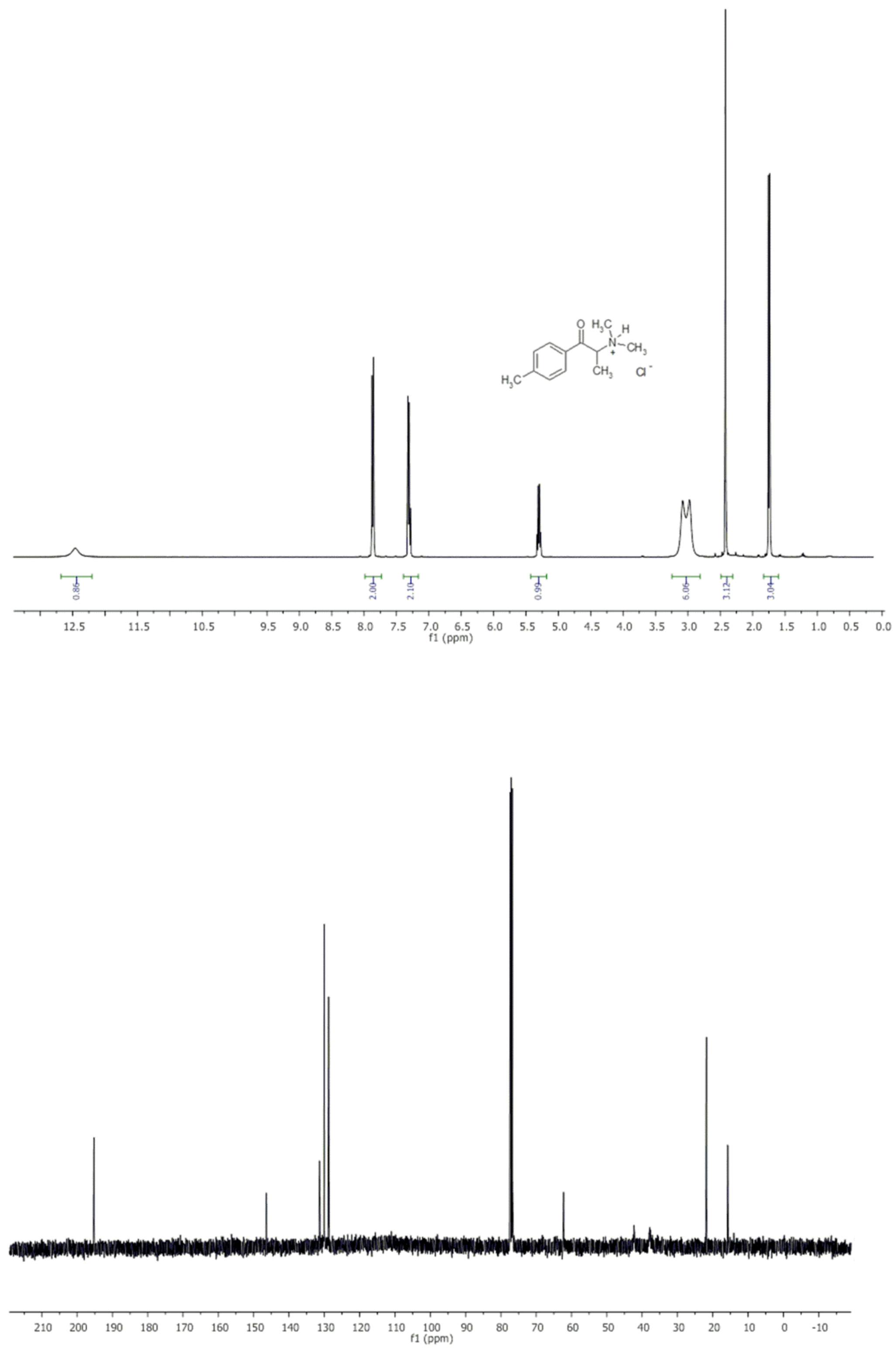


**Fig.S6** $^{1}H$ NMR ( *upper*) and $^{13}C$ NMR (*lower*) spectra of compound **2** in $CDCl_3$

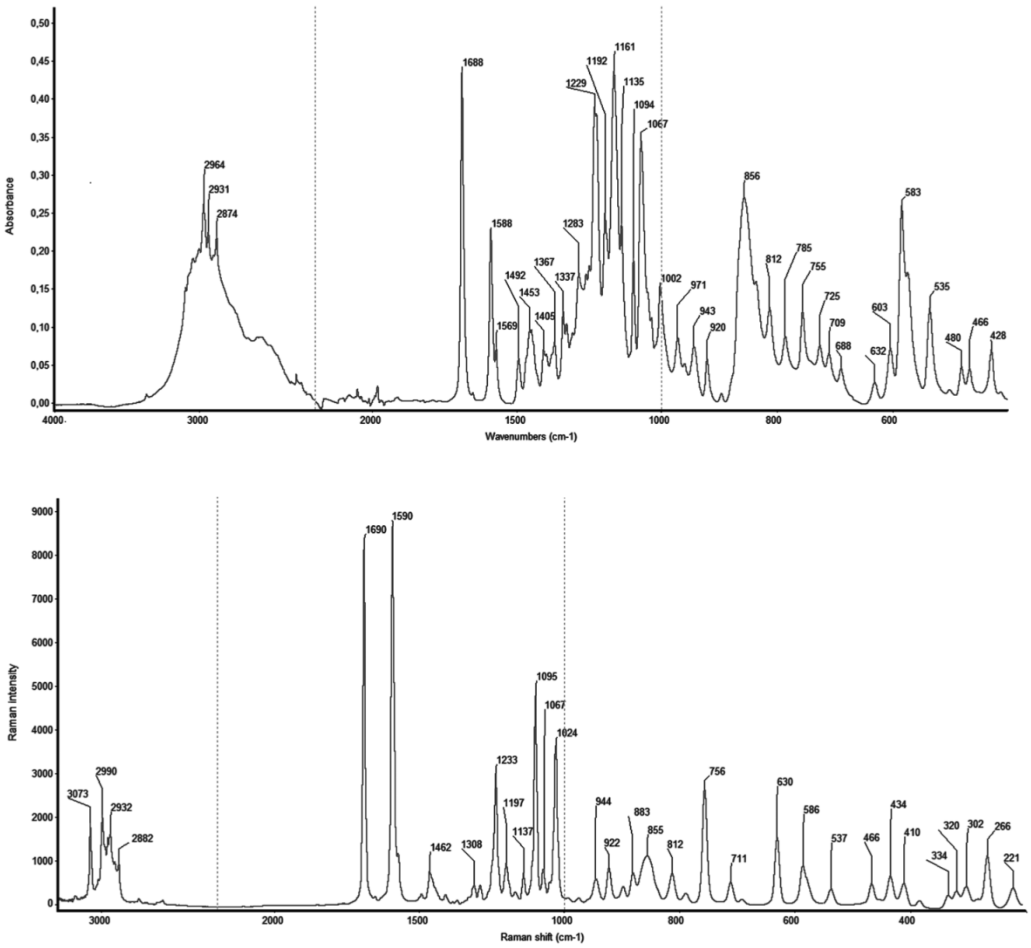


**Fig. S7** Infrared ( *upper*) and Raman (*lower*) spectra of compound **1** (4-chloro-α -PVP hydrogen sulfate)

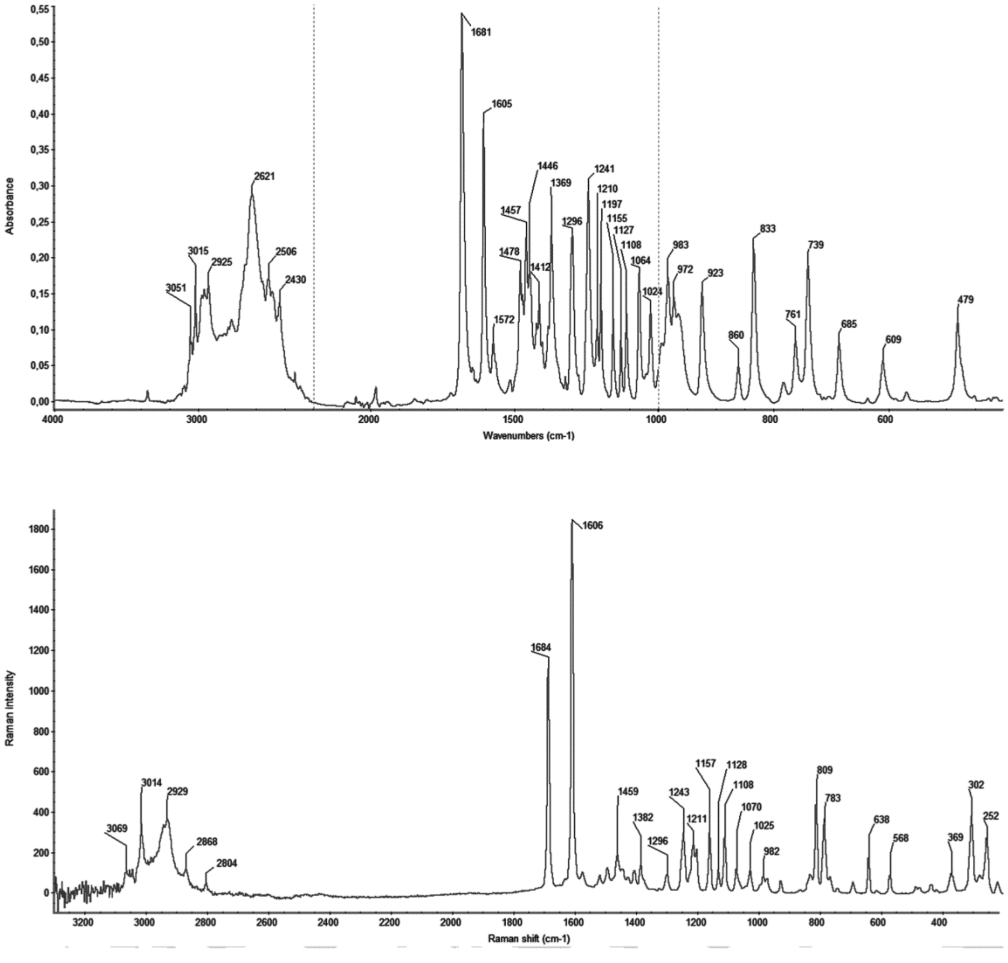


**Fig. S8** Infrared ( *upper*) and Raman (*lower*) spectra of compound **2** (4-MDMC hydrochloride)
